# Larval and/or Adult Exposure to Intraguild Predator *Harmonia axyridis* Alters Reproductive Allocation Decisions and Offspring Growth in *Menochilus sexmaculatus*

**DOI:** 10.3390/insects14060496

**Published:** 2023-05-25

**Authors:** Xinglin Yu, Rui Tang, Tongxian Liu, Baoli Qiu

**Affiliations:** 1 Engineering Research Center of Biological Control Ministry of Education the People’s Republic of China, South China Agricultural University, Guangzhou 510642, China; 2 State Key Laboratory of Crop Stress Biology for Arid Areas, College of Plant Protection, Northwest A&F University, Yangling 712100, China; 3 Key Laboratory of Integrated Pest Management on Crops in Northwestern Loess Plateau of Ministry of Agriculture, College of Plant Protection, Northwest A&F University, Yangling 712100, China; 4 Institute of Entomology, Guizhou University, Guiyang 550025, China; 5 Engineering Research Center of Biotechnology for Active Substances, Ministry of Education, Chongqing Normal University, Chongqing 401331, China

**Keywords:** *Menochilus sexmaculatus*, *Harmonia axyridis*, maternal provisioning, trophic eggs, offspring weight

## Abstract

**Simple Summary:**

Predation has significant impacts on a prey’s ecology and evolution. In many species, maternal effects can improve offspring survival in response to predation by altering resource allocation to young and reproducing larger offspring. While the perception of predation risk can vary according to a prey’s life stage, whether maternally experienced intraguild predation (IGP) risk during different life stages affects the maternal effects of predatory insects remains unknown. This study examined the influence of exposure to intraguild predators (*Harmonia axyridis* (Pallas) (Coleoptera: Coccinellidae)) during the larval and/or adult stages on reproductive decisions and offspring growth in *Menochilus sexmaculatus* (Fabricius). Although the exposure of *M. sexmaculatus* larvae and/or adults to IGP risk did not influence egg size, it increased offspring body size in IGP environments; however, their exposure to IGP risk increased trophic egg production. Because IGP is commonly observed in *M. sexmaculatus* and favours relatively larger protagonists, the IGP risk perceived by different stages of *M. sexmaculatus* induces maternal effects; this can be an adaptive survival strategy to defend against *H. axyridis*. This study is an essential step towards understanding the role of maternal effects on the survival of *M. sexmaculatus* in IGP scenarios and furthers our understanding of *M. sexmaculatus* population dynamics in the field.

**Abstract:**

Maternal effects can reduce offspring susceptibility to predators by altering resource allocation to young and reproducing larger offspring. While the perception of predation risk can vary according to a prey’s life stage, it is unclear whether maternally experienced intraguild predation (IGP) risk during different life stages influences the maternal effects of predatory insects. We investigated the influence of exposure to intraguild predators (*Harmonia axyridis* (Pallas) (Coleoptera: Coccinellidae)) during the larval and/or adult stages on reproductive decisions and offspring growth in *Menochilus sexmaculatus* (Fabricius). Independent of the life stage, when *M. sexmaculatus* females experienced IGP risk, their body weight and fecundity decreased, but the proportion of trophic eggs produced increased. However, egg mass, egg clutch number, and egg clutch size were not influenced by the treatment. Next, when offspring encountered *H. axyridis*, mothers experiencing IGP risk during the larval and/or adult stages could increase their offspring’s weight. Moreover, offspring in IGP environments reached a similar size as those with no-IGP environments when mothers experienced IGP risk during the larval and/or adult stages. Overall, *M. sexmaculatus* larval and/or adult exposure to IGP risk had no influence on egg size, but increased offspring body size when faced with *H. axyridis*. Additionally, mothers experiencing IGP risk during different life stages showed increased production of trophic eggs. Because IGP is frequently observed on *M. sexmaculatus* and favours relatively larger individuals, different stages of *M. sexmaculatus* express threat-sensitively to IGP risk; inducing maternal effects can be an adaptive survival strategy to defend against *H. axyridis*.

## 1. Introduction

Predation plays an important role in altering the ecology and evolution of prey [1,2]. In their struggle to reduce predation, prey exhibit anti-predator adaptations involving morphological defences, behavioural defences, and changes in life history traits [3,4,5,6]. In addition to the expression of anti-predator defences, mothers can adaptively help their offspring cope with predation stress [7]. Quite a few studies have documented that females experiencing predation increase their offspring’s body size [8,9,10,11,12], which can be generally advantageous for prey because some predator species have limited ability to handle large prey [13]. Such effects are usually dubbed maternal effects and are described as phenotypic effects of mothers changing the offspring phenotype but not genotype, playing an important role in the suitability of the next generation for future risky environments [9,12,14].

In oviparous species, the body size of offspring is usually related to the investment of resources in eggs, sometimes leading to changes in egg size [15,16]. In predation environments, mothers commonly produce eggs that are larger than usual [9]. Generally, larger-sized eggs contain extra resources that enable the offspring to have larger body sizes and better survival in response to predators [9,16]. Most evidence involving maternal investment in reproduction is derived from adult experience, possibly because female adults are often in direct contact with their offspring and the complicated environment that will be risky for their offspring [10,11]. However, prey species at different developmental stages of life may encounter and respond to predation risks variously [17]. This may be distinct among insects with complex life cycles, with discrete larval and adult stages exhibiting differences in morphology, behaviour, and physiology [11,17,18]. For example, in many insects, soft-bodied larvae have a higher probability of being vulnerable to attack than sclerotized adults [19]. Given the vulnerability of prey at the larval stage, the impact of predation pressure experienced in these stages may be carried over and become distinct in adults [20,21]. Previous studies have found that *Leptinotarsa decemlineata* (Say) (Coleoptera: Chrysomelidae), an herbivorous insect species, produces larger eggs when maternally exposed to predation risk during the larval stage, leading to an increase in offspring body size [11]. Maternal effects on reproductive allocation decisions to young and offspring growth may also be observed in predatory insects and other natural enemies when they perceive predation risks during the larval stage.

In natural communities, predatory insects are usually attacked by multiple predator species, such as heterospecific predators within a guild and higher-order natural enemies [22]. When sharing the same prey species, different predator species may compete for this prey and kill each other via intraguild predation (IGP) [17,23]. Intraguild predation is prevalent among predatory insect species [24], and its direction and frequency are affected by the relative body size of antagonistic individuals [17,18]. Generally, the young developmental stages, which are the small-sized individuals, are more vulnerable to relatively larger ones [17,18,25]. In this case, maternal effects can act as informative cues to buffer offspring from intraguild (IG) predators [7]. However, few studies explore whether maternally experienced IG predators during the larval and/or adult stages affect maternal provisioning of predatory insects. Moreover, it is unknown whether these mothers can increase their offspring’s body size in the presence of IG predators.

The present study investigated the ladybird species *Menochilus sexmaculatus* (Fabricius) (Coleoptera: Coccinellidae), which is an efficient enemy of many insect pests, such as *Aphis craccivora* Koch (Hemiptera: Aphididae) [26,27,28]. In the field, *M. sexmaculatus* is abundant throughout the year and interacts with other ladybird species within the guild, including *Harmonia axyridis* (Pallas) [29,30]. *Menochilus sexmaculatus* female adults are not at risk of IGP, but the larvae of *M*. *sexmaculatus* are susceptible to *H. axyridis* [31]. Thus, *M. sexmaculatus* females may maternally prepare their offspring to better manage future IGP risks. Previous studies have demonstrated that the egg size and reproductive output of *M. sexmaculatus* change in response to constraints enforced by female body size and age, and differently sized females alter their offspring’s weight with comparable effects at different ages [16]. However, it is unknown whether *M*. *sexmaculatus* females exposed to *H. axyridis* during the larval and/or adult stages will alter their reproductive decisions, and how their offspring’s growth changes when facing IGP risk.

In this study, we first tested whether maternal exposure to IGP risk during the larval and/or adult stages altered maternal investment in offspring. In addition, although previous studies on *L. decemlineata* have found that the consumption of trophic eggs did not alter the body size of offspring when predation risk was present, it could reduce the vulnerability of individuals to predation by improving their anti-predator responses [32]. Therefore, we also recorded the number of trophic eggs laid by *M*. *sexmaculatus*. We then assessed whether maternally experienced IGP risk during the larval and/or adult stages influenced offspring growth in IGP environments.

## 2. Materials and Methods

### 2.1. Plants and Insects

*Aphis craccivora* colonies were established from a laboratory colony at the South China Agricultural University (Guangzhou, Guangdong, China) during the summer of 2020, and maintained on broad bean plants (*Vicia faba* L., cultivar: Jinnong). The broad bean plants were grown in 12 cm diameter plastic pots filled with soil mix (peat moss: perlite = 3:1) and enclosed in ventilated cages (45 × 45 × 45 cm). *Menochilus sexmaculatus* were collected from common bean fields at the Experimental Farm in South China Agricultural University, and *H. axyridis* were obtained from a laboratory colony at Qingdao Agricultural University (Qingdao, Shandong, China). Both ladybird species were obtained in August 2021. A pair of *M. sexmaculatus* or *H. axyridis* were placed in 3 cm Petri dishes (diameter: 3 cm, height: 1.5 cm), and provided with *A. craccivora* as prey. *Menochilus sexmaculatus* or *H. axyridis* eggs laid within 24 h were collected and singly placed in a 3 cm Petri dish using a soft paintbrush. When the eggs hatched, *M. sexmaculatus* or *H. axyridis* larvae were maintained individually in 3 cm Petri dishes with *A. craccivora* daily until pupation. To prevent any induced effects of *H. axyridis* on *M. sexmaculatus*, we maintained these two ladybird colonies separately until the experiments were conducted (more than 10 generations). In addition, although both *H. axyridis* larvae and adults can generate an IGP risk in *M. sexmaculatus* [31], to simplify our experimental design, we only used virgin male adults in all subsequent experiments. All plants and insect colonies were kept in an air-conditioned room at 23 ± 1 °C, 65 ± 5% RH, and a 14:10 (L:D) h photoperiod.

### 2.2. Pre-Experimental Procedures

To generate *M. sexmaculatus* females experiencing IGP risk during different life stages for subsequent experiments, four treatments were designed. Siblings from the same egg clutch were allowed to experience IGP risk during the larval (yes/no) and adult (yes/no) stages, resulting in four groups of experimental females: (1) females never exposed to IGP risk; (2) females experiencing IGP risk only during the larval stage; (3) females experiencing IGP risk only during the adult stage; and (4) females experiencing IGP risk during both the larval and adult stages. We used siblings from the same clutch to control for variation among clutches (e.g., emergence time). A total of 20 clutches laid by different females were used, and their clutch identities were tracked throughout the experiment. For each clutch, the eggs were individually placed in a Petri dish with a water-saturated cotton ball.

When new hatchlings of *M. sexmaculatus* (<24 h old) began to disperse in the Petri dish, four hatchlings from the same clutch were randomly selected and individually placed in a nylon mesh enclosure (100 mesh or 0.149 mm screen, length: 11 cm, width: 9 cm), and each was reared with 5 mg of aphids with a broad bean leaf. Then, we allocated half of these nylon mesh enclosures to either the “risk” or “no risk” plastic cups. To generate “risk” cups, we introduced three male adults of *H. axyridis* into a plastic cup (diameter: 6.5 cm, height: 5 cm), and supplied 90 mg of *A. craccivora* as prey. A “no risk” cup only contained 90 mg of *A. craccivora*. In both “risk” and “no risk” cups, one broad bean leaf was added to feed the aphids. In addition, in both the nylon mesh enclosures and plastic cups, leaves were detached from 3- to 4-week-old broad beans, and each petiole was wrapped with a water-saturated cotton ball to prevent wilting. Additionally, all aphids, broad bean leaves, and cotton balls in the nylon mesh enclosure and plastic cups were refreshed daily. Moreover, as *M. sexmaculatus* grew through the larval stages, larvae were supplied broad bean leaves infested with an excess of *A. craccivora* (approximately 5, 10, 20, and 30 mg of *A. craccivora* were provided daily to the first, second, third, and fourth larval instar, respectively) until pupation.

Within 24 h of emergence, adults from each arena (with or without IGP risk) were sexed. Data was not collected for egg clutches when pupae did not emerge, or when the adults were male. Thus, we only used 12 clutches, giving rise to females for subsequent experiments. These newly emerged females were individually placed on a nylon mesh enclosure with 30 mg of *A. craccivora* daily along with the leaf of a broad bean. The moisture content of the leaves in the nylon mesh enclosure was maintained using a piece of wet cotton wool. For each of these 12 egg clutches, 2 females whose larval stage had either experienced or never experienced IGP risk were equally allocated to the next “risk” or “no risk” plastic cup. Both “risk” and “no risk” plastic cups were prepared using the procedure described above. Each adult female from all treatments was paired with an unrelated male (20–30 days old) that had never experienced an IGP risk. The mating process was allowed to last for 24 h in a new 3 cm Petri dish, which only contained a water-saturated cotton ball. This was conducted every 24 h at the time of prey refreshment to maintain the reproductive vigour of the females. To ensure female insemination, the procedure was repeated until the subsequent experiment started.

### 2.3. Oviposition Responses by M. sexmaculatus Females

To investigate whether *M. sexmaculatus* females experiencing IGP risk during the larval and/or adult stages influenced their reproductive decisions, eggs from the four treatments were collected in the second week after oviposition had started. The egg collection time was selected based on a previous study that found that *M. sexmaculatus* females typically have a production peak during the second week of their reproductive life span [33]. Before collecting the eggs, we measured female weight to the nearest 0.1 mg using an electronic balance (OHAUS CP214, Shanghai, China). Because such measurements were conducted before females started to experience peak oviposition, they might reflect differences in female conditions caused by exposure to *H. axyridis* during the larval and/or adult stages.

Clutches were collected at two different times in the second week after the oviposition of *M. sexmaculatus* had started: the first clutch (first to second days of the second week) or the second clutch (third to fourth days of the second week). In the first egg clutch, five randomly selected eggs (laid within 24 h) were weighed to the nearest 0.1 μg using a microbalance (Sartorious MSA 3.6P-000-DM, Gottingen, Germany). The eggs in the second clutch were used to determine the total number of eggs, egg clutch number, egg clutch size (number of eggs per clutch), and ratio of trophic eggs (number of trophic eggs/total number of eggs). Eggs that were undeveloped and were a pale-yellow colour were considered trophic eggs [34], because Osawa [35] established that these eggs did not hatch even when maintained for five days. Thus, the eggs in the second clutches were checked daily for five successive days to record the number of trophic eggs. In addition, to avoid cannibalism caused by hatching asynchrony, the eggs were individually placed in a Petri dish, which only contained a small piece of soaked cotton wool. Each treatment was repeated 12 times.

### 2.4. Offspring Growth

We examined whether females that experienced IGP risk during the larval and/or adult stages induced weight changes in their offspring. Two newly emerged first instar larvae (<24 h old) from each second egg clutch in the four treatment groups were randomly chosen and individually placed in a new Petri dish. When larvae began to disperse in the Petri dish, two larvae from one egg clutch were individually reared in a nylon mesh enclosure with 90 second- and third-instar *A. craccivora* nymphs with a broad bean leaf, and equally allocated to “risk” or “no risk” plastic cups. The “risk” and “no risk” plastic cups were prepared as previously described. The broad bean leaf in the nylon mesh enclosure was kept moist using a piece of soaked cotton wool, with water added using a dropper every 12 h. After 3 days, 4-day-old larvae were weighed to the nearest 0.1 mg using an electronic balance. Each treatment was repeated 12 times.

### 2.5. Statistical Analyses

All statistical analyses were performed using SPSS (version 21.0; SPSS, Inc., Chicago, IL, USA). For the oviposition responses by *M. sexmaculatus* assays, data on female body weight, egg weight, total egg number, egg clutch number, egg clutch size, and percentage of trophic eggs met the assumptions of normality and homoscedasticity, and were assessed using a one-way analysis of variance (ANOVA). Differences among treatments were determined using Tukey’s honestly significant difference (HSD) test (*p* < 0.05).

For offspring growth trials, because the raw data met the assumptions of normality and homoscedasticity, we used a two-way ANOVA to examine the main and interactive effects of life stage experience, and IGP risk on offspring weight. An independent sample *t*-test (*p* < 0.05) was used to compare the weight of offspring in IGP versus no-IGP scenarios when their mothers did not experience IGP risk or experienced IGP risk during the larval and/or adult stages. In addition, differences among offspring weight in IGP or no-IGP scenarios were determined using Tukey’s HSD test (*p* < 0.05). In all figures, the results were present as mean values ± standard error (SE).

## 3. Results

### 3.1. Oviposition Responses by M. sexmaculatus Females

**Figure 1:**
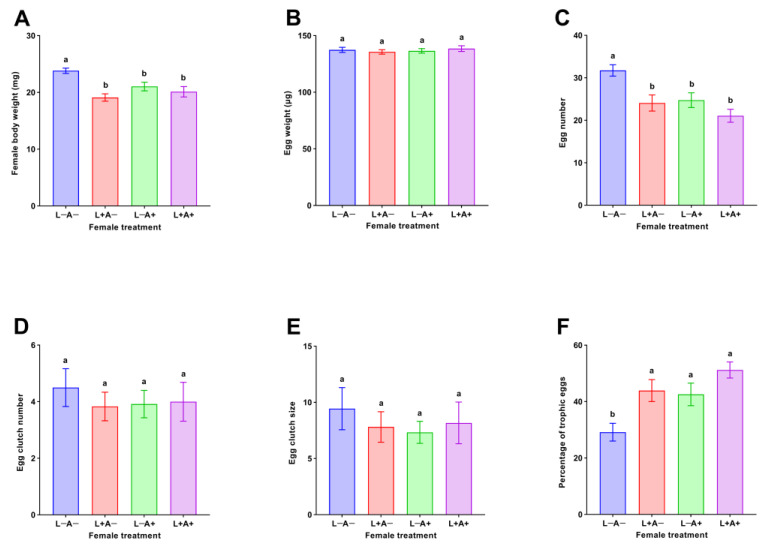
Effects of *Menochilus sexmaculatus* larval and/or adult exposure to intraguild predation (IGP) risk on female body weight (**A**), egg weight (**B**), egg number (**C**), egg clutch number (**D**), egg clutch size (**E**), and percentage of trophic eggs (**F**). L−A− = mothers never exposing to IGP risk, L+A− = mothers experiencing IGP risk only during the larval stage, L−A+ = mothers experiencing IGP risk only during the adult stage, and L+A+ = mothers experiencing IGP risk during both the larval and adult stages. Different letters indicate statistical differences between treatments (*p* < 0.05).

The body weight of *M. sexmaculatus* females differed significantly among the female treatments (*F*_3, 44_ = 8.002, *p* < 0.001). The female body weight of *M. sexmaculatus* was significantly heavier in trial with females that never experienced IGP risk than in the other three trials, including the trial with females that experienced IGP risk during the larval stage (Tukey’s HSD test: *p* < 0.001), trial with females that experienced IGP risk during the adult stage (Tukey’s HSD test: *p* = 0.042), and trial with females that experienced IGP risk during both the larval and adult stages (Tukey’s HSD test: *p* = 0.004). However, there were no significant differences in body weight of *M. sexmaculatus* females that experienced IGP risk during the larval stage, adult stage, and both the larval and adult stages (Tukey’s HSD test: *p* ≥ 0.238 for all) (Figure 1A). The egg weights were not significantly different among the four female treatments (*F*_3, 236_ = 0.329, *p* = 0.805) (Figure 1B).

The number of eggs laid by *M. sexmaculatus* females varied significantly when they experienced the four IGP risk scenarios (*F*_3, 44_ = 7.583, *p* < 0.001). More eggs were produced in the trial with females that never experienced IGP risk compared with the three other trials, including the trial with females that experienced IGP risk during the larval stage (Tukey’s HSD test: *p* = 0.010), trial with females that experienced IGP risk during the adult stage (Tukey’s HSD test: *p* = 0.021), and trial with females that experienced IGP risk during both the larval and adult stages (Tukey’s HSD test: *p* < 0.001). However, no significant differences were found in the number of eggs laid by *M. sexmaculatus* females among trials with females that experienced IGP risk during the larval stage, adult stage, and both the larval and adult stages (Tukey’s HSD test: *p* ≥ 0.399 for all) (Figure 1C). The egg clutch number (*F*_3, 44_ = 0.255, *p* = 0.857), and egg clutch size (*F*_3, 44_ = 0.333, *p* = 0.801) were similar among the four female treatments (Figure 1D,E).

The percentage of trophic eggs laid by *M. sexmaculatus* females varied significantly when females experienced different IGP risk scenarios (*F*_3, 44_ = 6.804, *p* = 0.001). The proportion of trophic eggs from females that did not experience IGP risk was significantly lower than that from females that experienced IGP risk during different life stages: during the larval stage (Tukey’s HSD test: *p* = 0.024), during the adult stage (Tukey’s HSD test: *p* = 0.048), and during both the larval and adult stages (Tukey’s HSD test: *p* < 0.001). However, there were no significant differences in the percentage of trophic eggs laid by *M. sexmaculatus* females among trials with females that experienced IGP risk during the larval stage, adult stage, and both the larval and adult stages (Tukey’s HSD test: *p* ≥ 0.318 for all) (Figure 1F).

### 3.2. Offspring Growth

The IGP risk (*F*_1, 88_ = 9.253, *p* = 0.003) and the interaction between life stage experience and IGP risk significantly affected offspring weight (*F*_3, 88_ = 11.213, *p* < 0.001). However, life stage experience had insignificant effects on larval weight (*F*_3, 88_ = 0.049, *p* = 0.985).

When offspring grew without exposure to IGP risk, the offspring were significantly heavier in the trial with females that never experienced IGP risk compared with those in other trials including the trial with mothers that experienced IGP risk during the larval stage (Tukey’s HSD test: *p* = 0.002), trial with mothers that experienced IGP risk during the adult stage (Tukey’s HSD test: *p* = 0.011), and trial with mothers that experienced IGP risk during both the larval and adult stages (Tukey’s HSD test: *p* = 0.011). Additionally, there were no significant differences in offspring weight among trials with females that experienced IGP risk during the larval stage, adult stage, and both the larval and adult stages (Tukey’s HSD test: *p* ≥ 0.942 for all). In contrast, when offspring grew with exposure to IGP risk, the offspring weight was significantly lower in the trial with females that never experienced IGP risk than those in other trials including the trial with mothers that experienced IGP risk during the larval stage (Tukey’s HSD test: *p* = 0.010), trials with mothers that experienced IGP risk during the adult stage (Tukey’s HSD test: *p* = 0.010), and trials with mothers that experienced IGP risk during both the larval and adult stages (Tukey’s HSD test: *p* = 0.019). In addition, no significant differences were found in the offspring weight among trials with females that experienced IGP risk during the larval stage, adult stage, and both the larval and adult stages (Tukey’s HSD test: *p* ≥ 0.994 for all).

**Figure 2:**
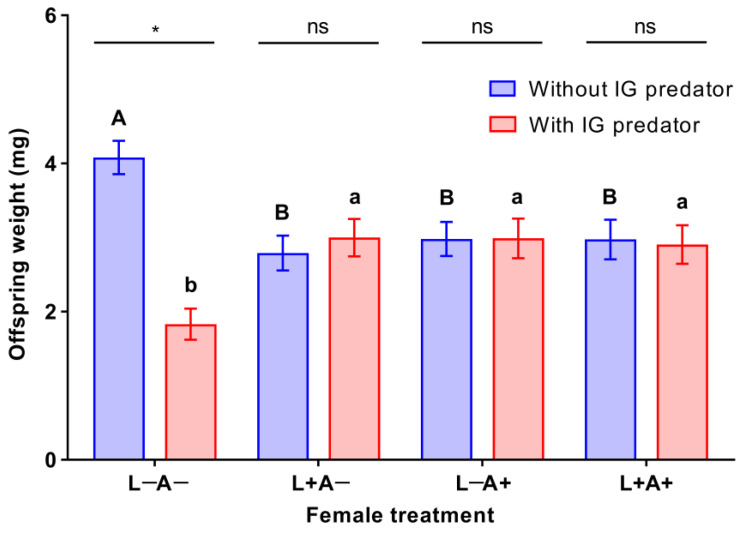
The weight of offspring in trials conducted with or without intraguild predators (*Harmonia axyridis*), when *Menochilus sexmaculatus* mothers experienced intraguild predation (IGP) during the larval and/or adult stages. L−A− = mothers never exposed to IGP risk, L+A− = mothers experiencing IGP risk only during the larval stage, L−A+ = mothers experiencing IGP risk only during the adult stage, and L+A+ = mothers experiencing IGP risk during both the larval and adult stages. Bars with different capital or lowercase letters within the treatments with or without IGP risk, respectively, are significantly different (*p* < 0.05). Within each female treatment, ‘*’ represents significant differences (*p* < 0.05) and ‘ns’ stands for not significantly different between the treatments with and without IGP risk.

When mothers never experienced IGP risk, the offspring were significantly heavier in the trail where offspring grew without exposure to IGP risk compared with those in the trial where offspring grew with exposure to IGP risk (*t*_22_ = 7.305, *p* < 0.001). In contrast, when mothers experienced IGP risk during the larval and/or adult stages, no significant differences were observed in the weight of offspring between the IGP and no-IGP scenarios (*t*_22_ = −0.605–0.179, *p* = 0.552–0.981) (Figure 2).

## 4. Discussion

Intraguild predation is common among insect predators [24]. However, the magnitude and direction of IGP are influenced by the size of the protagonists, and in most cases, larger protagonists win confrontations [17,18,25]. To decrease the risk of IGP in offspring, intraguild prey (IG prey) mothers can help their offspring via selecting optimal sites for oviposition, and/or killing the potential offspring IG predators [7,36]. Apart from these methods, there are few studies exploring the effects of maternally experienced IGP risk during the larval and/or adult stages on reproductive allocation decisions, and subsequent offspring growth in response to IGP risk among predatory insect species. In this study, we found that exposure to IGP risk during the larval and/or adult stages significantly reduced the maternal body mass and number of eggs laid, but increased the proportion of trophic eggs of *M. sexmaculatus*. However, egg mass, egg clutch number, and egg clutch size of *M. sexmaculatus* were not affected by the treatment. In addition, when offspring were confronted with IG predators, the weight of offspring from mothers that experienced IGP risk during the larval and/or adult stages was significantly heavier than those from unexperienced mothers. Moreover, when mothers experienced IGP risk during the larval and/or adult stages, the weight of the offspring was not significantly different between the trials with or without IGP risk.

The present study revealed that *M. sexmaculatus* females experiencing IGP risk had lower weights and produced fewer eggs than unexperienced females. However, the risk of IGP experienced by *M. sexmaculatus* females did not influence the egg size. Previous studies have found that IG prey reduce the prey consumption when IG predators or their cues are present [37]. Accordingly, a reduction in feeding in *M. sexmaculatus* larvae and/or adults may occur when they are exposed to *H. axyridis* adults, and ultimately elicit body mass reduction in *M. sexmaculatus* female adults. In insects, smaller females generally lay smaller and fewer eggs than larger females [16,35]. However, apart from body size, the ovariole number are also the main determinants for egg size and number, and the number of ovarioles in individuals is positively correlated with female body size [35,38]. Notably, with a decrease in ovariole number, the egg number was reduced, but the egg size increased [39,40,41]. In the current study, we suggest that smaller-sized females of *M. sexmaculatus*, in relation to environmental conditions with intense IGP, may have fewer ovarioles. Given that female *M. sexmaculatus* may adjust their egg size in response to both the number of ovarioles and body size, possible reductions in ovariole number in *M. sexmaculatus* may increase their egg size to compensate for the potential reduction in egg size associated with small maternal body size. Consequently, independent of the maternal life stage, the effect of IGP-risk-induced maternal effects on *M. sexmaculatus* reproduction could only be detected in egg number, but not in egg size.

Additionally, *M. sexmaculatus* females that experienced IGP risk during the larval and/or adult stages laid a higher percentage of trophic eggs. Eggs of this type could not develop because of the failure of their mothers to fertilize them [32,42]. These eggs are usually laid as a highly nutritious first meal for the new hatchlings, and play an important role in increasing the offspring’s survival, fitness, and anti-predator responses [32,43,44]. A previous study on *L. decemlineata* indicated that mothers that experienced predation risk during the adult stage had an increased percentage of trophic eggs. Cannibals that fed on one egg (viable or trophic) after hatching exhibited increased anti-predator responses and reductions in predation chances up to *c*. 30% [32]. In this study, the adaptive function of *M. sexmaculatus* in promoting trophic egg production may also be regarded as an effectively maternal investment for offspring to enhance their chances of survival in response to IGP risk.

Some studies have found that mothers can produce larger-sized eggs containing more resources, which enable the offspring to have a larger body size and higher survival chances in response to predators [9,11,16]. However, our results showed that although the egg size was not affected by the mother’s experience, *M. sexmaculatus* offspring from mothers that experienced IGP risk had heavier weights than those from unexperienced mothers in trials when *H. axyridis* was present. One possible explanation is that risk-induced maternal effects not only provide nutritional benefits, but also offer hormones to their offspring [9,32]. Previous studies have suggested that three-spined stickleback (*Gasterosteus aculeatus* L.) females exposed to the risk of predation during egg formation could improve the anti-predator defences of offspring, which might be mediated through exposure to maternally derived cortisol [9]. Notably, variation in the content of hormones in the egg may occur independently of egg size [45,46]. Accordingly, we suggest that when confronted with *H. axyridis*, *M. sexmaculatus* females may cause egg-size-independent increases in the content of hormones in eggs, and improve the ability of defences against *H. axyridis* by increasing offspring body size. This mechanism may also explain why the offspring in IGP scenarios reached a similar size as those in no-IGP scenarios when their mothers experienced IGP risk during the larval and/or adult stages. However, we observed that when both mothers and offspring were exposed to no IGP risk, the offspring were significantly heavier compared with those of offspring in assays with mothers that experienced IGP risk and offspring that grew without exposure to IGP risk. In general, maternal effects can help organisms elude predation risk not only by increasing offspring body size, but also in many other ways [9,47,48,49]. It is possible that *M. sexmaculatus* females experience IGP risk that prepares other defence mechanisms to enhance the offspring’s ability to defend against potential future IG predators by inducing subtle changes in the content of resources in their eggs that are not detectable by egg size alone, but at the cost of growth and life history traits in offspring.

Here, we found that *M. sexmaculatus* females not only provide resources fed to their offspring, but also can increase the offspring body size. Yet we found no differences in terms of egg size, proportion of trophic eggs, and offspring body size when *M. sexmaculatus* females experienced IGP risk during the larval stage, adult stage, and both the larval and adult stages. In the field, the niches of *M. sexmaculatus* and *H. axyridis* can overlap for several generations in similar crops [30]. Because this environment is fairly stable over time, both the IGP risk experienced by females during their own larval stage and/or the presence of potential offspring IG predators during the actual oviposition may convey information to mothers about the IGP risk for their offspring. Possibly, unknown factors in the long-term coexistence might have caused threat sensitivity to IGP risk in both the larvae and adults of *M. sexmaculatus*, and thus have resulted in an adaptive maternal effect. Given that IGP may commonly occur between *M. sexmaculatus* and *H. axyridis*, the ability to increase body size is important for improving *M. sexmaculatus* offspring survival in IGP environments. Thus, both the larval and adult stages of *M. sexmaculatus* behave threat-sensitively to IGP risk, inducing maternal effects that can be an adaptive survival strategy for *M. sexmaculatus* to cope with IGP risk.

## 5. Conclusions

Our results confirmed that larvae and/or adults of *M. sexmaculatus* exposed to IGP risk had no impact on the size of the eggs, but increased the body size of their offspring in response to *H. axyridis*. However, mothers experiencing IGP risks during the larval and/or adult stages can increase the production of undeveloped trophic eggs. In *Sancassania berlesei* (Michael) (Acari: Acaridae), maternal effects arise by variations in reproductive allocation to young individuals, which affect individual life histories, leading to marked population dynamic effects that propagate over several generations [45]. Further studies are required to elucidate whether *H. axyridis*-induced maternal effects on reproductive allocation decisions and offspring growth in *M. sexmaculatus* would influence their long-term population dynamics. Additionally, since our study found an increase in the production of trophic eggs, the effects of trophic egg consumption on offspring anti-predator defences in response to IG predators should be investigated in future studies.

## Data Availability

The data presented in this study are available on request from the corresponding author.
